# Soil Water Availability Changes in Amount and Timing Favor the Growth and Competitiveness of Grass Than a Co-dominant Legume in Their Mixtures

**DOI:** 10.3389/fpls.2021.723839

**Published:** 2021-10-22

**Authors:** Weizhou Xu, Xiping Deng, Bingcheng Xu, Jairo A. Palta, Yinglong Chen

**Affiliations:** ^1^College of Life Sciences, Yulin University, Yulin, China; ^2^State Key Laboratory of Soil Erosion and Dryland Farming on the Loess Plateau, Northwest A&F University, Yangling, China; ^3^Institute of Soil and Water Conservation, Chinese Academy of Sciences and Ministry of Water Resources, Yangling, China; ^4^School of Agriculture and Environment, The UWA Institute of Agriculture, The University of Western Australia, Perth, WA, Australia; ^5^CSIRO Agriculture and Food, Wembley, WA, Australia

**Keywords:** growth period, soil water availability, biomass production, competition, mixture ratio

## Abstract

The grasslands on the semi-arid Loess Plateau of China are expected to be particularly responsive to the size and frequency changes of extreme precipitation events because their ecological processes are largely driven by distinct soil moisture pulses. However, the plant growth and competitiveness of co-dominant species in response to the changes in the amount and timing of soil water are still unclear. Thus, two co-dominant species, *Bothriochloa ischaemum* and *Lespedeza davurica*, were grown in seven mixture ratios under three watering regimes [80 ± 5% pot soil capacity (FC) (high watering), 60 ± 5% FC (moderate watering), and 40 ± 5% FC (low watering)] in a pot experiment. The soil water contents were rapidly improved from low to moderate water and from moderate to high water, respectively, at the heading, flowering, and maturity stages of *B. ischaemum*, and were maintained until the end of the growing season of each species. The biomass production of both species increased significantly with the increased soil water contents, particularly at the heading and flowering periods, with a more pronounced increase in *B. ischaemum* in the mixtures. The root/shoot ratio of both species was decreased when the soil water availability increased at the heading or flowering periods. The total biomass production, water use efficiency (WUE), and relative yield total (RYT) increased gradually with the increase of *B. ischaemum* in the mixtures. The relative competition intensity was below zero in *B. ischaemum*, and above zero in *L. davurica*. The competitive balance index calculated for *B. ischaemum* was increased with the increase of the soil water contents. *Bothriochloa ischaemum* responded more positively to the periodical increase in soil water availability than *L. davurica*, indicating that the abundance of *B. ischaemum* could increase in relatively wet seasons or plenty-rainfall periods. In addition, the mixture ratio of 10:2 (*B. ischaemum* to *L. davurica*) was the most compatible combination for the improved biomass production, WUE, and RYTs across all soil water treatments.

## Introduction

Rainfall variability greatly affects the structure and function of ecosystems globally, especially in the arid and semi-arid regions (Knapp et al., 2003; Post and Knapp, 2020). The response of the plant community to rainfall fluctuations is an integrative outcome from each species, particularly the dominant species. Thus examining their responses to altered rainfall patterns can provide a better understanding and prediction of community changes (Schwinning et al., 2004; Báez et al., 2013). The eco-physiological response of dominant species to the changes in rainfall pattern in the arid and semi-arid areas, not only depends on the capability of drought resistance in individual species, but also on the ability to grow and recover after rainfalls, largely influenced by the antecedent soil water availability (Niu et al., 2016; Xiong et al., 2017). Plant biomass allocation, water use strategies, and plant-plant interactions (either facilitation or competition) are the key integrative measures of the ecosystem function, which have been the major focus in plant eco-physiology research over the last decade (Soliveres et al., 2012; Xu et al., 2013; Foxx and Florian, 2019). In the Inner Mongolian region of north China, the stability of primary productivity in grassland communities is high, mainly because there is a strong compensatory effect among the performance of species under fluctuant rainfall conditions (Bai et al., 2004). It is likely that the intensity and outcome of plant-plant interactions may change with the soil water availability. Therefore, intraspecific competition may replace interspecific facilitation as water stress is alleviated (García-Cervigón et al., 2013; Adler et al., 2018). Such shift in the competitive intensities and compensatory effect among species might be the key coexistence mechanism for stabilizing the grassland community productivity when encountering drought stress (Grant et al., 2014).

Grasslands play important roles in both the economy and ecology of the semi-arid region on the Loess Plateau of China, which is one of the most sensitive ecosystems to adapt to the changes in rainfall pattern during the growing season in water-limited regions (Fu et al., 2010; Lee et al., 2014; Bhandari et al., 2015). The construction of artificial grasslands with high regional adaptation was an effective practice to address the problems created by grassland degradation, and it is the key measure to ensure the sustainable development of grasslands in the region (Shan and Xu, 2009; Xu et al., 2011a). In addition, the use of grass and legume mixtures has been widely proposed in this region because legumes fix nitrogen (N) into the rhizosphere, and can ameliorate the microsite in terms of nutrient availability (Xu et al., 2011b). Thus, the introduction of legumes at suitable mixture ratios into gramineous plants for establishing artificial grasslands is beneficial for improving biomass production, water use, and ecosystem stability, because there are complementary and mutually reinforcing roles between them (Xu et al., 2011b, 2013). Although the role of the strategies of biomass allocation and the specific responses of species in grassland communities to soil water availability have been highlighted in previous studies (Padilla et al., 2009; Niu et al., 2011; Soliveres et al., 2013; Xiong et al., 2017), detailed information on these strategies and specific responses of grasslands with mixed grass and legume to the amount and timing of soil water changes is scarce. With the advent of climate change, rainfall has become the most important driver of biological activity in the arid and semi-arid regions, and the assessment of the specific responses of co-occurring species to rainfall pulses would favor the prediction of changes in ecological processes and the vulnerability of grassland ecosystems (Fay, 2009).

However, construction of artificial grasslands in this region is currently limited by the lack of appropriate grass species and its single grassland structure, with low eco-adaptation to local environments (Shan and Xu, 2009). Moreover, the response of the grassland ecosystem to the altered rainfall patterns is largely driven by the dominant native plant species in the communities (Schwinning et al., 2004; Xiong et al., 2017). *Bothriochloa ischaemum* (L.) Keng and *Lespedeza davurica* (Laxm.) Schindl are two co-occurring native species widely distributed across semi-arid grassland communities. *Bothriochloa ischaemum* is a perennial herbaceous grass, while *L. davurica* is a perennial leguminous subshrub. The two species have great eco-adaptation to the regional environment and climate and play important roles in reducing soil and water loss and maintaining distinctive natural landscapes in the area. Understanding how these co-occurring native species respond to altered water availability in mixtures will provide information on their coexistence mechanisms and will aid in the selection of appropriate measures in establishing artificial grasslands. Our previous studies evaluated the biomass, competitive ability, and water use efficiency (WUE) of *B. ischaemum* and *L. davurica* under three constant water regimes and four fertility treatments (Xu et al., 2011a, b, 2013). The results showed that there exist complementary effects between both species when growing together, with the biomass relative yield total (RYT) values of their mixtures being generally greater than 1. We also investigated the root morphological traits of *B. ischaemum* when mixed with *L. davurica* under soil watering treatments, which showed that the soil moisture availability at the early growing season strongly increased its root growth (Wang et al., 2018).

The focus of the current study was on the response of co-occurring species to the changes in soil water availability because the changes in rainfall patterns with the advent of climate change are expected to cause more frequent extreme rainfalls and longer intervals between events (Liu et al., 2012; Niu et al., 2016). Knowledge about the biomass allocation strategy and relative competitive ability of both species in response to varying mixture ratios and watering regimes is still unknown. Therefore, the aims of the present study were: (1) to evaluate the responses of the biomass allocation and water use of both species to the changes in the amount and timing of soil watering availability, (2) to identify the plant-plant interactions in response to varying mixture ratios and watering regimes, and (3) to determine which mixture ratio of the two species is the most suitable for artificial grasslands based on their interacting coexistence mechanisms.

## Materials and Methods

### Plant Materials and Growth Conditions

The seeds of both species were collected in the autumn of 2011 from an experimental field at the Ansai Research Station (ARS) of the Chinese Academy of Sciences (CAS), located at the semi-arid hilly-gully region on the Loess Plateau (36°51′N, 109°19′E). The experiment was conducted in pots at the nursery of the State Key Laboratory of Soil Erosion and Dryland Farming on the Loess Plateau, Yangling, Shaanxi Province, China (34°12′N, 108°7′E), which has a mean annual temperature of 12.9°C, a maximum mean monthly temperature of 26.7°C in July, a minimum temperature of −1 to −2°C in January, and a mean annual rainfall of 637.6 mm.

The loessial soil used in the experiment was collected from the 0–20 cm soil layer in the natural grassland at ARS. The soil organic matter content was 3.6 g kg^–1^, and the total nitrogen (N), phosphorus (P), and potassium (K) contents were 2.5, 6.6, and 1.9 g kg^–1^, respectively. The soil available N, P, and K contents were 19.62, 50.78, and 101.55 mg kg^–1^, respectively, and the soil pH value was 8.37. The soil gravimetric moisture content at field capacity and wilting point were 20 and 4%, respectively.

To ensure a sufficient nutrient supply for the plants, N and P were applied in each pot as basic fertilizers in the forms of CON_2_H_4_ (0.48 g) and KH_2_PO_4_ (3.95 g), respectively, and both were mixed uniformly with the soil during potting. Each cylindrical plastic pot (20 cm diameter, 30 cm height) was filled with 9 kg of the air-dried soil, and an open-end plastic pipe (2 cm diameter) was inserted inside each pot adjacent to the inner wall to supply water at about 10 cm from the pot bottom. During rainy days, all the pots were covered by a rainout shelter.

### Species Combination Design

A designed method of replacement series was used as reported by Xu et al. (2011b). The two species were grown at seven mixture ratios (0:12, 2:10, 4:8, 6:6, 8:4, 10:2, and 12:0) of *B. ischaemum* to *L. davurica* with a density of 12 plants per pot on April 1, 2012. The seeds of each species were initially sown in 12 equally spaced holes, and in each hole, three to five seeds per species were dibbled. The schems of plant arrangement of both species in different mixture ratios per hole was followed as given by Xu et al. (2011b). Immediately after emergence, the seedlings were thinned to one plant per dibble, i.e., to 12 plants per pot. All the pots were initially well-watered to the field capacity during the seedling establishment till the tillering period of *B. ischaemum.*

### Watering Treatment

**FIGURE 1:**
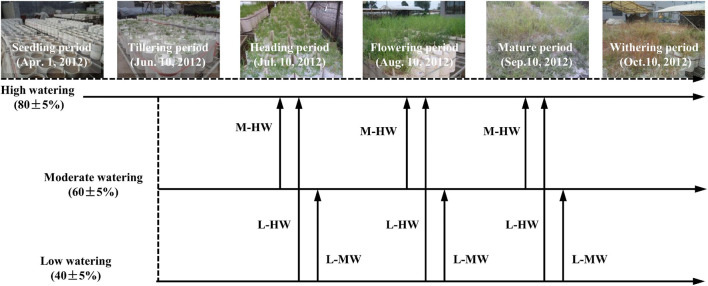
The scheme of periodical soil watering treatments based on the growth periods of *Bothriochloa ischaemum*. High Watering (HW), 80 ± 5% pot soil capacity (FC); Moderate Watering (MW), 60 ± 5% FC; Low Watering (LW), 40 ± 5% FC. Moderate to high watering (M-HW), soil water contents increased from MW to HW regime; Low to moderate or high watering (L-MW or L-HW), soil water contents increased from LW to MW or HW regime, respectively.

In the semi-arid loess hilly-gully region on the Loess Plateau of China, *B. ischaemum* was the dominant species while *L. davurica* was the subordinate species in the natural grassland community (Xu et al., 2013). Their growth change and response to rainfall event variations greatly affected their co-existent community, thus in this study, the initiation of watering and re-watering treatments were carried out on the four key phonological periods of *B. ischaemum*, i.e., the tillering, heading, flowering, and mature period (Figure 1).

The watering treatments were initiated when seedlings of *B. ischaemum* had five leaves and *L. davurica* was still under branching. All the pots were divided into three groups on June 10, 2012, and each group was exposed to a watering regime as follows:

(1) High watering: maintain pot soil capacity (FC) at 80 ± 5% (HW).
(2) Moderate watering: maintain pots at FC of 60 ± 5% (MW).
(3) Low watering: maintain pots at FC of 40 ± 5% (LW).

Then, three re-watering treatments were carried out during the three growth periods as follows: the heading period (July 10, 2012), flowering period (August 10, 2012), and mature period (September 12, 2012) of *B. ischaemum*. In each vegetative stage, the soil water contents were increased from MW to HW regime (referred to as M-HW), increased from LW to HW regime (referred to as L-HW), and increased from LW to MW regime (referred to as L-MW) through re-watering, respectively (Figure 1).

For the nursing of the desired water regimes, plant transpiration was measured by weighing each pot every day at 18:00 h, and each pot was watered *via* the plastic pipes to maintain the target water regime. To reduce soil compaction caused by watering and soil evaporation, 40 g of perlite was spread on the soil surface of each pot. The levels of the soil water content after re-watering were maintained until the end of the growing season, on October 10, 2012.

There were a total of 420 pots: 7 (mixture ratios) × 12 [3 (re-watering) × 3 (growth period) + 3 (no re-watering)] × 5 (replications). All the pots were arranged in a completely random design within the replications for each treatment.

### Shoot and Root Biomass Production

At the end of the growth season on October 10, 2012, all the pots of each treatment were harvested and the shoot and root parts of each species were separated. The soil from roots was removed by washing with water and the shoot and root biomass were determined after drying at 75°C for 48 h in an air-dried oven. The root/shoot ratio (RSR) was calculated as the ratio of root to shoot dry biomass.

### Water Use Efficiency

The daily evapotranspiration was obtained by weighing the pots daily at 18:00 h. Three identical pots filled with soil but without plants for each water treatment were used to estimate the soil evaporation, and they were watered individually to the desired regime as those with plants. The estimate of the soil evaporation was subtracted from evapotranspiration to calculate the transpiration of both species. So, WUE was defined as the amount of the total biomass produced per unit volume of the water transpired.

### Competitive Indices

Several indicators for species interaction have been used in intercropping research. These indicators can be divided into three categories: quantification of competition intensity, the effect of competition, and the outcome of competition (Weigelt and Jolliffe, 2003). Relative competition intensity (RCI) and competitive balance (CB) were employed for studying the intensity of competition, and RYT was employed for analyzing the competition effects. These indexes have been commonly used in experimental designs involving series planting replacements, and they were calculated from the dry biomass of each species and their various proportions within the replacement series.

Relative competition intensity was used to compare the intra- and interspecific competition of the two species (Sammul et al., 2000). The equal intra- and interspecific competition are indicated by RCI = 0, and a positive value indicates that the interspecific competition is high, while a negative value means high intraspecific competition. The RCI was calculated as follows:


(1) $$RCI=\left(Y_{BB}\times Z_{BL}-Y_{BL}\right)/\left(Y_{BB}\times Z_{BL}\right)$$

where *Y*_*BB*_ is the biomass production of *B. ischaemum* in monoculture and Y*_*LL*_* is the biomass production of *L. davurica* in monoculture. *Y*_*BL*_ or Y*_*LB*_* is the biomass production of *B. ischaemum* or *L. davurica* in their mixtures. *Z*_*BL*_ or Z*_*LB*_* is mixture proportions of *B. ischaemum* or *L. davurica* in their mixtures.

Competitive balance was another index that was used to quantify the relative competitive ability of each species in the mixtures (Wilson, 1988). A CB of 0 indicates no competition or equal competitive abilities between the two species, and a positive value indicates that *B. ischaemum* has a higher competitive ability than *L. davurica*, while a negative value indicates that *L. davurica* was the better competitor. The CB was calculated as follows:


(2) $$CB=l\text{n}\left(Y_{BL}\times Y_{LB}\right)$$

The relative yield total was estimated to assess the biological efficiency of the intercropping system (Williams and Mccarthy, 2001). If the RYT = 1, then the two species have equal demands for the same limiting resource. Values greater than 1 indicate that the mixtures are advantageous for biomass production compared with monocultures, while less than 1 indicates mutual antagonism. The RYT was calculated as follows:


(3) $$RYT=Y_{BL}/Y_{BB}+Y_{BL}/Y_{LL}$$

### Statistical Analysis

In the study, each treatment had five repetitions, and three replications of each treatment were randomly selected for sampling. The data were checked using the normality (Shapiro-Wilk’s test) and homogeneity of variances (Levene’s test) before analysis and were expressed as the mean ± SE of the three replications. The differences in the mean values of the plant biomass, WUE, RSR, and each competitive indices were compared among treatments using a one-way ANOVA followed by Tukey’s honestly significant difference (HSD) tests, and the statistical significance was set at *P* < 0.05. Three-way ANOVA was used to evaluate the interactive effects of soil watering, growth period, and mixture ratio on biomass production, WUE, RSR, and competitive indices (RYT, RCI_*B*_, RCI_*L*_, and CB values) of the species. The least significant differences (LSD) (*P* = 0.05) were calculated for the mean separations and are shown in the figures. All statistical analyses of the data were conducted using SPSS 17.0 (IBM, Armonk, NY, United States).

## Results

### Biomass Production

**FIGURE 2:**
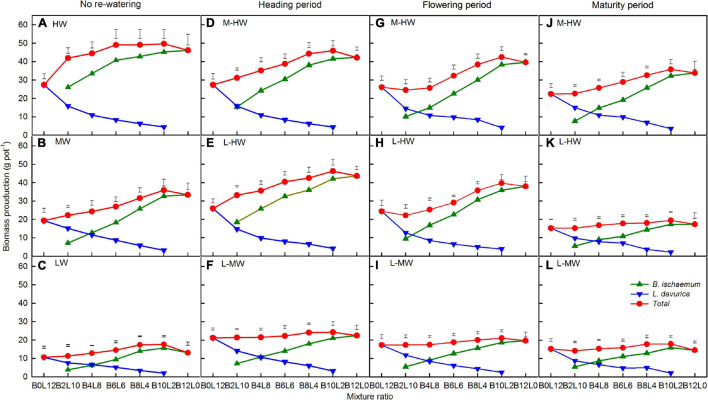
**(A–L)** The total biomass production of *B. ischaemum* (B) and *Lespedeza davurica* (L) under the soil watering treatments during different growth periods within the replacement series. B*i*L*j* (*i, j* = 0, 2, 4, 6, 8, 10, 12; *i* + *j* = 12) in abscissa indicates the plant numbers of *B. ischaemum* to *L. davurica* in the mixtures; Vertical bars represent least significant difference (LSD) 0.05 values. Refer to Figure 1 for the codes of the water treatments.

Increasing the soil water availability, growth period, mixture ratio, and their interactions significantly affected the total biomass production (Table 1). The total biomass production in each species was significantly increased as the proportion of *B. ischaemum* was increased in the mixture ratios, and the highest biomass production was at the 10:2 mixture ratio in most cases (Figure 2). Among the seven mixtures, *B. ischaemum* showed a higher biomass production at the 10:2 mixture ratio and in the monoculture (i.e., 12:0), while the *L. davurica* monoculture had a higher biomass production (Figure 2).

**TABLE 1:** Effects of soil watering, growth period, mixture ratio, and their interaction in the three-way ANOVA on the total biomass production and water use efficiency (WUE) of both species.

**Source of variation**	**d*f***	**Total biomass production**		**Water use efficiency**	
		***F***	***P***	***F***	***P***
Soil watering (SW)	2	2,638.659	**<0.001**	113.215	**<0.001**
Growth period (GP)	2	1,924.063	**<0.001**	34.844	**<0.001**
Mixture ratio (MR)	6	386.590	**<0.001**	509.001	**<0.001**
SW × GP	4	271.068	**<0.001**	38.017	**<0.001**
SW × MR	12	54.662	**<0.001**	13.156	**<0.001**
GP × MR	12	24.561	**<0.001**	13.570	**<0.001**
SW × GP × MR	24	8.520	**<0.001**	6.321	**<0.001**

*Probabilities considered statistically significant are indicated in bold form.*

Under each watering regime, *B. ischaemum* had a higher biomass production than *L. davurica* at the equal mixture ratio (i.e., 6:6), except when re-watering from L-MW at the heading and maturity periods (Figure 2). The curves of the replacement series diagrams of biomass production in *B. ischaemum* were concave, while those in *L. davurica* were convex, and their lines intersected to the left of the 6:6 mixture ratio, *i*.*e*., the proportion of *B. ischaemum* was lower in mixtures (Figure 2).

Compared with MW, the average total biomass production across all mixture ratios was increased by about 40.4 and 18.3% when the soil water increased from M-HW at the heading and flowering periods, respectively (Figure 2). Those values were significantly increased by about 21.7–166.1% when the soil water increased from L-HW at each period and significantly increased by about 60.2 and 33.1% when the soil water increased from L-MW at the heading and flowering stages, compared with the LW treatment (Figure 2).

### Root/Shoot Ratio

**FIGURE 3:**
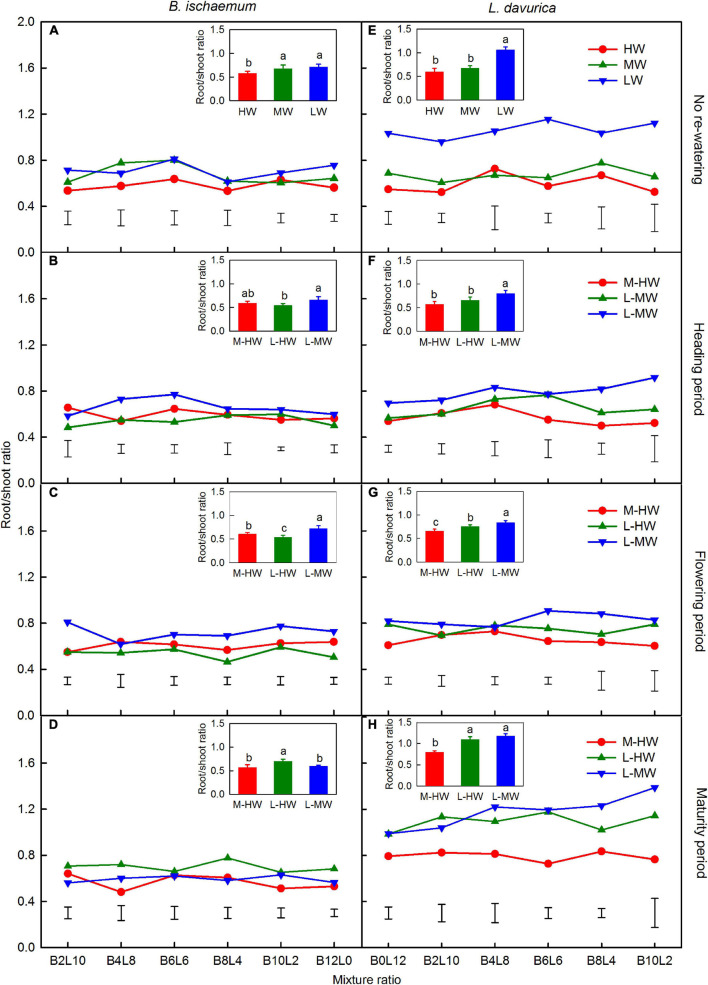
**(A–H)** The root/shoot ratio (RSR) of *B. ischaemum* (B) and *L. davurica* (L) under the soil watering treatments during different growth periods within the replacement series. B*i*L*j* (*i, j* = 0, 2, 4, 6, 8, 10, 12; *i* + *j* = 12) in abscissa indicates the plant numbers of *B. ischaemum* to *L. davurica* in the mixtures; Vertical bars represent LSD 0.05 values. The values in the small histogram are the mean across all mixture ratios, and the columns and bars indicate the mean ± SE, the letters are used for the comparisons between different water treatments by LSD test (*P* < 0.05). Refer to Figure 1 for the codes of the water treatments.

Increasing the soil water availability, growth period, mixture ratio, and their interactions significantly affected the RSR of both species, except for the effect of the growth period (Table 2). There were no significant changes in the RSR of both species across the different mixture ratios under the watering regimes, but the increase in soil water availability significantly decreased the RSR in most cases (Figure 3). The RSR in *B. ischaemum* ranged from 0.46 to 0.81 across mixture ratios under watering regimes, while the RSR in *L. davurica* ranged from 0.50 to 1.39 (Figure 3).

**TABLE 2:** Effects of soil watering, growth period, mixture ratio, and their interaction in the three-way ANOVA on the biomass production and root/shoot ratio (RSR) of both species.

**Source of variation**	**d*f***	***Bothriochloa ischaemum***				***Lespedeza davurica***			
		**Biomass production**		**Root/shoot ratio**		**Biomass production**		**Root/shoot ratio**	
		***F***	***P***	***F***	***P***	***F***	***P***	***F***	***P***
Soil watering (SW)	2	520.772	**<0.001**	35.333	**<0.001**	230.583	**<0.001**	167.754	**<0.001**
Growth period (GP)	2	394.826	**<0.001**	2.485	0.088	220.371	**<0.001**	355.237	**<0.001**
Mixture ratio (MR)	5	399.734	**<0.001**	3.764	**0.003**	1978.953	**<0.001**	4.974	**<0.001**
SW × GP	3	119.527	**<0.001**	65.012	**<0.001**	37.833	**<0.001**	25.060	**<0.001**
SW × MR	10	19.353	**<0.001**	3.678	**<0.001**	18.774	**<0.001**	6.083	**<0.001**
GP × MR	10	7.123	**<0.001**	5.590	**<0.001**	25.439	**<0.001**	2.707	**0.005**
SW × GP × MR	15	2.197	**0.010**	3.311	**<0.001**	7.198	**<0.001**	1.913	**0.029**

*Probabilities considered statistically significant are indicated in bold form.*

For *B. ischaemum*, the average RSR decreased by about 10.3–16.2% when the soil water increased from M-HW compared with MW (Figure 3). Compared with LW, those values decreased by 5.1 and 18.7% under MW and HW, decreased by 1.7–24.5% when the soil water increased from L-HW and decreased by 7 and 16.7% when the soil water increased from L-MW, respectively, at the heading and maturity stages (Figure 3).

For *L. davurica*, the average RSR was decreased by 15.8 and 3.2% when the soil water increased from M-HW at the heading and flowering stages, compared with the MW level (Figure 3). Compared with LW, the RSR was decreased by 21.5–38.4% when the soil water increased from L-MW and -HW at the heading and flowering stages, respectively, and increased by 11–17.5% when soil water increased from L-MW and -HW at the maturity stage (Figure 3).

### Relative Yield Total

**FIGURE 4:**
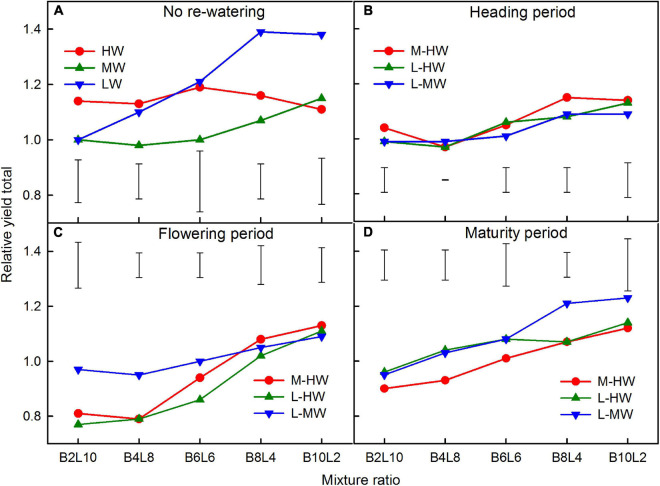
**(A–D)** The relative yield total (RYT) for the total biomass production of *B. ischaemum* (B) and *L. davurica* (L) in the mixtures under the soil watering treatments during different growth periods. B*i*L*j* (*i, j* = 0, 2, 4, 6, 8, 10, 12; *i* + *j* = 12) in abscissa indicates the plant numbers of *B. ischaemum* to *L. davurica* in their mixtures; Vertical bars represent LSD 0.05 values. Refer to Figure 1 for the codes of the water treatments.

Increasing the soil water availability, growth period, mixture ratio, and their interactions significantly affected the RYT in the mixtures, except for the effect of the interaction of the soil watering and mixture ratio (Table 3). The RYT significantly decreased with the increase in the soil water availability but increased gradually as the proportion of *B. ischaemum* increased in the mixtures, except under HW (Figure 4). The average RYT across all water treatments at the 2:10 and 4:8 mixture ratios were all less than 1, indicating that there was a similar mutual antagonism between both species in such mixture ratio, while the relatively higher values obtained at the 10:2 mixture ratio, indicate that the mixed planting of both species in the 10:2 mixture ratio was more advantageous for biomass production compared with monocultures (Figure 4).

**TABLE 3:** Effects of soil watering, growth period, mixture ratio, and their interaction in the three-way ANOVA on the relative yield total and relative competition intensity calculated from the biomass of both species, and the competitive balance index calculated from the total biomass, shoot biomass, and root biomass of *Bothriochloa ischaemum.*

**Source of variation**	**d*f***	**Relative yield total**		**Relative competition intensity**				**Competitive balance index**					
				***Bothriochloa ischaemum***		***Lespedeza davurica***		**Total**		**Root**		**Shoot**	
		***F***	***P***	***F***	***P***	***F***	***P***	***F***	***P***	***F***	***P***	***F***	***P***
Soil watering (SW)	2	7.268	**0.001**	0.485	0.617	22.355	**<0.001**	19.077	**<0.001**	16.042	**<0.001**	8.488	**<0.001**
Growth period (GP)	2	37.866	**<0.001**	95.926	**<0.001**	5.340	**0.006**	28.320	**<0.001**	9.769	**<0.001**	31.017	**<0.001**
Mixture ratio (MR)	4	47.163	**<0.001**	222.902	**<0.001**	64.976	**<0.001**	2900.387	**<0.001**	1790.973	**<0.001**	1886.749	**<0.001**
SW × GP	3	3.931	**0.011**	28.574	**<0.001**	34.099	**<0.001**	60.228	**<0.001**	46.405	**<0.001**	36.840	**<0.001**
SW × MR	8	0.615	0.763	0.803	0.601	6.643	**<0.001**	5.943	**<0.001**	3.709	**0.001**	4.424	**<0.001**
GP × MR	8	2.096	**0.044**	16.457	**<0.001**	5.081	**<0.001**	9.404	**<0.001**	3.834	**0.001**	8.499	**<0.001**
SW × GP × MR	12	1.597	0.106	3.976	**<0.001**	8.203	**<0.001**	9.845	**<0.001**	5.920	**<0.001**	7.483	**<0.001**

*Probabilities considered statistically significant are indicated in bold form.*

Compared with the RYT under MW, the average RYT was decreased by 8.7 and 3.3% when the soil water increased from M-HW at the flowering and maturity periods, respectively, but increased by 2.9% when the soil water increased from M-HW at the heading period (Figure 4). Compared with LW, those values decreased by 9.8–25.4% when the soil water increased from L-MW or -HW at each period (Figure 4).

### Relative Competition Intensity

**FIGURE 5:**
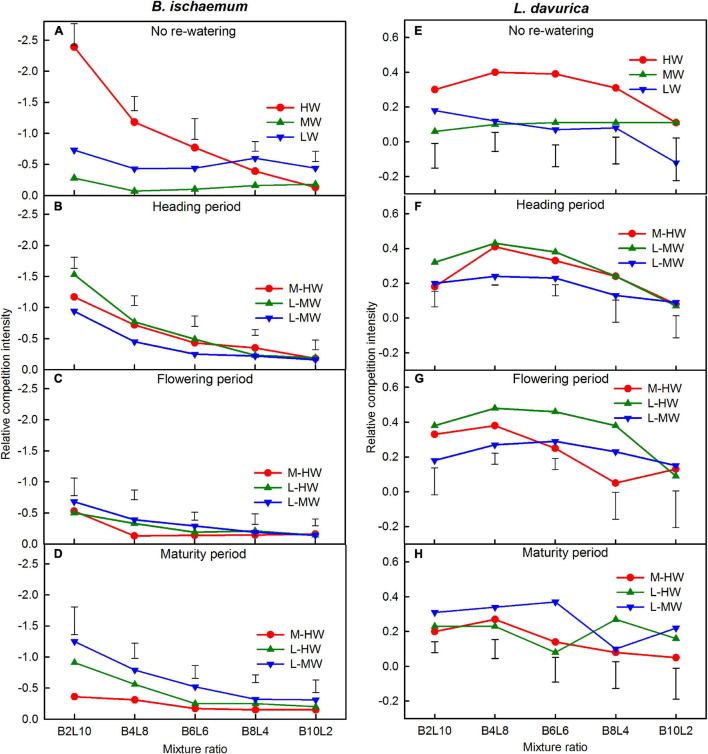
**(A–H)** The relative competition intensity (RCI) for the total biomass of *B. ischaemum* (B) and *L. davurica* (L) in the mixtures under the soil watering treatments during different growth periods. B*i*L*j* (*i, j* = 0, 2, 4, 6, 8, 10, 12; *i* + *j* = 12) in abscissa indicates the plant numbers of *B. ischaemum* to *L. davurica* in their mixtures; Vertical bars represent LSD 0.05 values. Refer to Figure 1 for the codes of the water treatments.

Increasing the soil water availability, growth period, mixture ratio, and their interactions significantly affected the RCI in the mixtures, except for the effect of the interactions of the soil watering and mixture ratio (Table 3). The RCI value of *B. ischaemum* was negative, indicating that the effects of the intraspecific competition were stronger than the interspecific competition. The RCI of *L. davurica* was greater than zero, indicating that the interspecific competition was stronger than the intraspecific competition (Figure 5). Under each watering regime, the RCI of both species was increased gradually as their proportions increased in the mixtures (Figure 5).

For *B. ischaemum*, the average RCI decreased by 44.3–260.8% when the soil water increased from M-HW, compared with MW (Figure 5). Compared with LW, those values were decreased by 21.6 and 20.8% when the soil water increased from L-HW at the heading and maturity periods, respectively, but increased by 17.8–70.1% when the soil water increased from L-MW and -HW (Figure 5).

For *L. davurica*, the average RCI values increased by 48–128% when the soil water availability increased from M-HW (Figure 5). Compared with LW, the RCI values were increased by 48.5–411.4% when the soil water increased from L-MW or -HW (Figure 5).

### Competitive Balance

**FIGURE 6:**
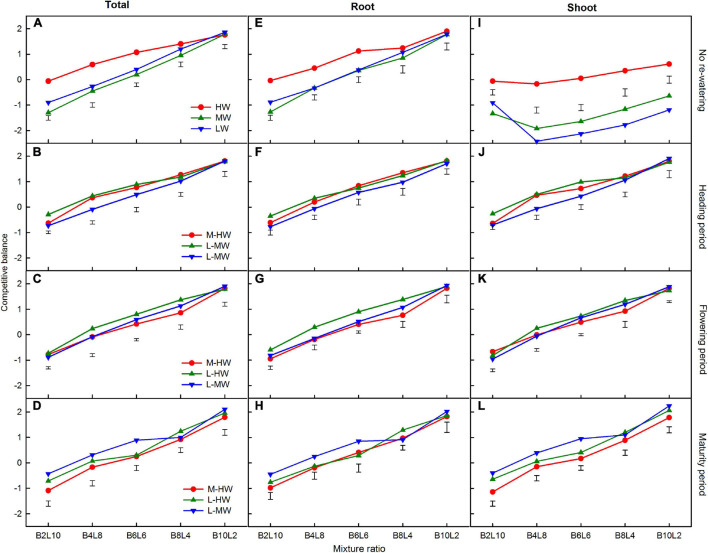
**(A–L)** The competitive balance index (CB) was calculated from the total biomass, shoot biomass, and root biomass production of *B. ischaemum* (B) in the mixtures under the soil watering treatments during different growth periods. B*i*L*j* (*i, j* = 0, 2, 4, 6, 8, 10, 12; *i* + *j* = 12) in abscissa indicates the plant numbers of *B. ischaemum* to *L. davurica* in their mixtures; Vertical bars represent LSD 0.05 values. Refer to Figure 1 for the codes of the water treatments.

Increasing the soil water availability, growth period, mixture ratio, and their interactions significantly affected the CB of *B. ischaemum* when calculated from the total, shoot, or root biomass (Table 3). The CB of *B. ischaemum* calculated from the total, shoot, or root biomass increased gradually as its proportion increased in the mixtures, and the relatively higher values were obtained at the 10:2 mixture ratio, indicating that the competitiveness of *B. ischaemum* was stronger (Figure 6).

The CB of *B. ischaemum* calculated from the total, shoot, or root biomass was significantly increased under increased soil water availability, especially when calculated based on the shoot biomass (Figure 6). The average CB values in *B. ischaemum* increased up to 2.04 times when calculated from the total biomass, ranged from 20.6 to 153.5% when calculated from the shoot biomass, and ranged from 0 to 160.1% when calculated from the root biomass (Figure 6).

### Water Use Efficiency

**FIGURE 7:**
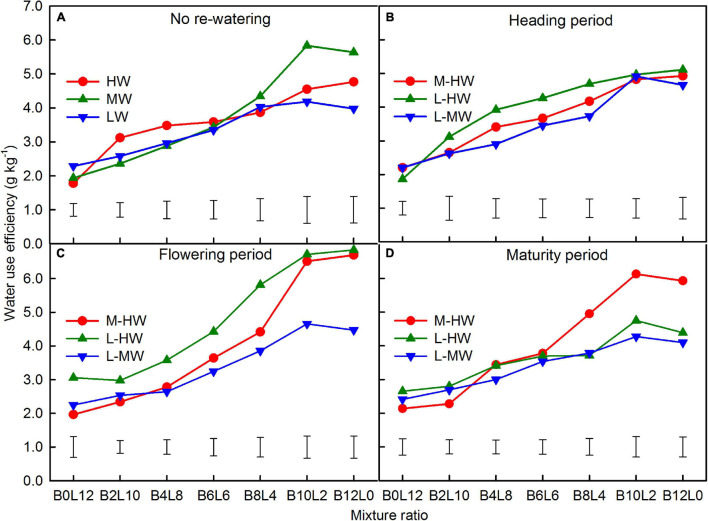
**(A–D)** The water use efficiency (WUE) based on the total biomass production and water transpired by *B. ischaemum* (B) and *L. davurica* (L) under the soil watering treatments during different growth periods within the replacement series. B*i*L*j* (*i, j* = 0, 2, 4, 6, 8, 10, 12; *i* + *j* = 12) in abscissa indicates the plant numbers of *B. ischaemum* to *L. davurica* in the mixtures; Vertical bars represent LSD 0.05 values. Refer to Figure 1 for the codes of the water treatments.

Increasing soil water availability, growth period, mixture ratio, and their interactions significantly affected the WUE of both species (Table 1). The WUE was significantly increased as the proportion of *B. ischaemum* increased while *L. davurica* decreased in the mixture ratios (Figure 7). Across all the watering regimes, the WUE was the highest at the 10:2 mixture ratio and *B. ischaemum* under monoculture, while the lowest WUE was in *L. davurica* under monoculture (Figure 7).

The WUE increased under the increasing soil water availability, and the average WUE across all mixtures ratios and in both species increased by 0–8.6% when the soil water increased from M-HW at each period (Figure 7). Compared with LW levels, the corresponding values increased by 0–43.0% when the soil water increased from L-MW or HW (Figure 7).

## Discussion

### Biomass Allocation and Water Use in Response to Soil Watering

Climate change has a strong impact on the availability and variability of water resources. It is expected that climate change will alter the seasonal distribution, frequency, and intensity of rainfall events in the semi-arid regions, which may result in fewer and heavier rainfall events (IPCC, 2014; Miao et al., 2016). In this study, the increasing soil water availability during the growing season improved the biomass of two co-occurring species of *B. ischaemum* and *L. davurica*, but larger increases occurred in the perennial herbaceous grass *B. ischaemum*, which largely contributed to the total biomass production when in mixtures (Figure 2). Increasing the soil water availability and planting mixture affected the biomass of both species, but the biomass production of *B. ischaemum* in the mixtures at the heading and flowering periods was much higher than in the monoculture, while *L. davurica* showed opposite patterns (Figure 2). This indicates that there was a positive additive effect of periodical re-watering and planting mixture on the biomass production of *B. ischaemum* during its early growing season (Xu et al., 2013; Wang et al., 2018). For *L. davurica*, the promoting effect of re-watering on its biomass can be suppressed by mixed planting and due to that, its growth was restrained in the mixtures (Xu et al., 2011a, b). Therefore, it can be predicted that the abundance and biomass of *B. ischaemum* will significantly increase in relatively wet seasons, years, or regions, compared with *L. davurica* (Peng et al., 2013; Niu et al., 2016).

Previous research indicated that mixed planting enhanced the photosynthetic performance of *B. ischaemum* in the heading and flowering stages, while those of *L. davurica* were improved only in the mature stage (Xu et al., 2014, 2017). In both species at the heading and flowering periods, the total biomass production was higher when the soil water increased from L-HW than increased from L-MW, but there were no significant differences between the soil water increased from M-HW and L-HW. These indicate that the re-watering intensity or increase in soil water availability from lower antecedent soil water availability were larger, leading to the greater community biomass production of the two species, especially at the early growing season (Xiong et al., 2017; Wang et al., 2018).

Increasing the soil water availability reduced the RSR in both species, with a smaller RSR value in *B. ischaemum* than in *L. davurica*. This was due to the synchronous increase in the shoot and root biomass, and such difference may enhance the competitive ability of *B. ischaemum* for above- and belowground resources under increasing soil water availability (Liu et al., 2012; Foxx and Florian, 2019). However, there was no clear changing trend in the biomass allocation of both species among mixture ratios under each water regime, indicating that both species maintained relatively stable root and shoot biomass allocation patterns in response to the altered mixture ratio and soil water regimes (Xu et al., 2011a; Zhang et al., 2017).

In the intercropping systems, the WUE was influenced by the plant density and species proportion (Xu et al., 2013; Ren et al., 2017). In this study, the WUE was increased as the proportion of *B. ischaemum* increased in the mixtures, and the highest WUE was obtained at the 10:2 mixture ratio of *B. ischaemum* and *L. davurica* (Figure 7). Across the mixture ratio and water regimes, the highest WUE was obtained in the 10:2 mixture ratio of *B. ischaemum* to *L. davurica*, which indicated that there may exist more mutual beneficial effects between the two species (Lithourgidis et al., 2011; Xu et al., 2013), and such mixture ratio is recommended for the use of *B. ischaemum* and *L. davurica* in the artificial grassland constructions in the region.

### Plant–Plant Interactions in Response to Varying Mixture Ratios

Ecological theory predicts that productivity will be higher in mixtures if species use environmental resources differently in both time or space (Banik et al., 2000; Wilsey, 2010). The RYT gives an accurate assessment of the greater biological efficiency of the intercropping situation (Williams and Mccarthy, 2001). The average RYT across the water regimes at the 6:6, 8:4, and 10:2 mixture ratios were 1.04–1.15 (Figure 4). The results indicate that mixed planting will improve the efficient use of land and environmental resources if the proportion of *B. ischaemum* is greater than 50% in the mixtures (Gao et al., 2009; Lithourgidis et al., 2011). Similar studies on legume and non-legume intercropping systems have given RYT values greater than 1, which have been attributed to the differences in the root and shoot characteristics, and the use of different N sources (Lithourgidis et al., 2011; Manasa et al., 2020). Results have shown that the RYT of both species in the mixed planting systems were greatly affected by their mixture ratios than the increasing soil water availability (Table 3 and Figure 4), which indicated that the ratio of the proportion of both species within the replacement series could affect the efficiency of intercropping systems significantly (Aynehband and Behrooz, 2011).

According to our previous studies, the intersection point of both biomass curves at the 6:6 mixture ratio in the replacement series diagram, suggests the equal competitive abilities between both species (Xu et al., 2011a, 2013). As shown in Figure 2, the biomass production curves of the two species intersect to the left of the 6:6 mixture ratio under each water treatment, indicating that *B. ischaemum* was more competitive than *L. davurica* in most cases, which also showed the frequency of the occurrence of interspecific competition between the two species in the mixtures (Foxx and Florian, 2019). Our results suggest that the growth of *L. davurica* appeared to be adversely affected by the competition from *B. ischaemum*, likely because the latter was more competitive in the mixtures under periodical soil water availability (Xu et al., 2011a, b).

Moreover, the species with positive CB values indicate its dominance and higher competitive ability than the component species in the intercropping systems (Wilson, 1988; Ghosh, 2004), thus, *B. ischaemum* was the dominant species with higher competitiveness when its proportion was greater than 50% in the mixtures with *L. davurica* (Figure 6). The higher competition of the grass over the legume species is likely to be explained by its extensive fine root system (Eekeren et al., 2009; Xu et al., 2011b). Moreover, the results obtained indicated the intensification in the shoot competition of *B. ischaemum* under high watering, which was ascribed to the increased CB values calculated from the shoot biomass (Figure 6). This was consistent with previous studies showing that the shoot competition of grassland species was more intense than the root under higher water availability (Lamb et al., 2007). The CB values of *B. ischaemum* increased as its proportion in the mixtures increased, indicating that its dominance was improved as its density was promoted (Dolijanovic et al., 2013; Yu et al., 2016).

The strong intraspecific competition in *B. ischaemum* and high interspecific competitive pressure in *L. davurica* indicates that the growth of *L. davurica* was restrained in the mixtures (Jolliffe et al., 1984; Xu et al., 2013). The gradual increase in the RCI in both species across all the watering regimes as their proportions increased in the mixtures, suggests that *B. ischaemum* faced lower intraspecific competitive pressure as its ratio increased in mixtures (García-Cervigón et al., 2013). Previous research also showed that the intraspecific competition between individuals of *B. ischaemum* was higher than the interspecific competition, and the competition between both species for soil P became intense when the soil N availability increased (Xu et al., 2013). The RCI values of both species decreased to zero as the proportions of *B. ischaemum* increased in mixtures, and the more stable ratio of the two species was obtained at 10:2 as their intra-and interspecific competitiveness tended to be equal (Jolliffe et al., 1984; Geijzendorffer et al., 2011).

## Conclusion

The biomass production, WUE, and competitive ability of the two native species in controlled mixtures were affected by changes in soil water availability. Soil watering at the early growth period promoted the biomass of *B. ischaemum*, and this herbaceous grass, hence, has given a higher contribution to the biomass production in the mixtures. The dominant position and competitiveness of *B. ischaemum* in the mixtures was increased with soil watering as supported by the positive CB values. The results clearly showed that *B. ischaemum* was the dominant species, and its proportion affected the complementarity effect of the two species in the mixtures. Furthermore, our results provided increasing evidence that *B. ischaemum* responded more positively to the increasing soil water availability than *L. davurica*, and perhaps ultimately, the abundance of this grass species in the region could increase in relatively wet seasons, years, or if rainfalls become more frequent in the future with climate change. The 10:2 proportion for intercropping *B. ischaemum* with *L. davurica* could be recommended as the most efficient species individuals combination for establishing artificial grasslands in the region when using those two native species, in terms of the higher biomass production and WUE, and the higher RYT and lower RCI values.

## Data Availability Statement

The original contributions presented in the study are included in the article/supplementary material, further inquiries can be directed to the corresponding author.

## Author Contributions

WX, XD, and BX conceived and designed the experiments. WX conducted the experiments. WX and BX analyzed the data and wrote the manuscript with major inputs from all co-authors. JP and YC provided critical comments and revised the manuscript. All authors contributed to the article and approved the submitted version.

## Conflict of Interest

The authors declare that the research was conducted in the absence of any commercial or financial relationships that could be construed as a potential conflict of interest.

## Publisher’s Note

All claims expressed in this article are solely those of the authors and do not necessarily represent those of their affiliated organizations, or those of the publisher, the editors and the reviewers. Any product that may be evaluated in this article, or claim that may be made by its manufacturer, is not guaranteed or endorsed by the publisher.
